# Thromboembolic and bleeding complications in patients with oesophageal cancer

**DOI:** 10.1002/bjs.11665

**Published:** 2020-05-19

**Authors:** F. I. Mulder, A. Hovenkamp, H. W. M. van Laarhoven, H. R. Büller, P. W. Kamphuisen, M. C. C. M. Hulshof, M. I. van Berge Henegouwen, S. Middeldorp, N. van Es

**Affiliations:** ^1^ Department of Vascular Medicine Amsterdam Cardiovascular Science Amsterdam the Netherlands; ^2^ Department of Radiotherapy, Amsterdam UMC University of Amsterdam Amsterdam the Netherlands; ^3^ Departments of Medical Oncology Amsterdam the Netherlands; ^4^ Surgery, Cancer Centre Amsterdam, Amsterdam UMC University of Amsterdam Amsterdam the Netherlands; ^5^ Department of Internal Medicine, Tergooi Hospitals Hilversum the Netherlands

## Abstract

**Background:**

In patients who undergo curative treatment for oesophageal cancer, risk estimates of venous thromboembolism (VTE), arterial thromboembolism and bleeding are needed to guide decisions about thromboprophylaxis.

**Methods:**

This was a single‐centre, retrospective cohort study of patients with stage I–III oesophageal cancer who received neoadjuvant chemoradiation followed by oesophagectomy. The outcomes VTE, arterial thromboembolism, major bleeding, clinically relevant non‐major bleeding and mortality were analysed for four consecutive cancer treatment stages (from diagnosis to neoadjuvant chemoradiotherapy, during neoadjuvant treatment, 30‐day postoperative period, and up to 6 months after postoperative period).

**Results:**

Some 511 patients were included. The 2‐year survival rate was 67·3 (95 per cent c.i. 63·2 to 71·7) per cent. During the 2‐year follow‐up, 50 patients (9·8 per cent) developed VTE, 20 (3·9 per cent) arterial thromboembolism, 21 (4·1 per cent) major bleeding and 30 (5·9 per cent) clinically relevant non‐major bleeding. The risk of these events was substantial at all treatment stages. Despite 30‐day postoperative thromboprophylaxis, 17 patients (3·3 per cent) developed VTE after surgery. Patients with VTE had worse survival (time‐varying hazard ratio 1·81, 95 per cent c.i. 1·25 to 2·64). Most bleeding events occurred around the time of medical intervention, and approximately one‐half during concomitant use of prophylactic or therapeutic anticoagulation.

**Conclusion:**

Patients with oesophageal cancer undergoing neoadjuvant chemoradiotherapy and surgery are at substantial risk of thromboembolic and bleeding events throughout all stages of treatment. Survival is worse in patients with thromboembolic events during follow‐up.

## Graphical abstract

A total of 511 patients who underwent curative chemoradiotherapy and subsequent tumour resection for oesophageal cancer were reviewed retrospectively for bleeding events and arterial or venous thromboembolism (VTE). During the 2‐year follow‐up, 50 patients (9·8 per cent) developed VTE, 20 (3·9 per cent) arterial thromboembolism, 21 (4·1 per cent) major bleeding and 30 (5·9 per cent) clinically relevant non‐major bleeding.

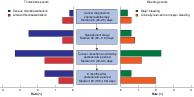

Perhaps inevitable

## Abstract (es)

**Antecedentes:**

Para tomar decisiones en cuanto a la profilaxis tromboembólica, es preciso estimar el riesgo de tromboembolismo venoso (*venous thromboembolism*, VTE), de tromboembolismo arterial y de hemorragia en pacientes a los que se vaya a realizar un tratamiento curativo para el cáncer de esófago.

**Métodos:**

Se realizó un estudio de cohortes retrospectivo de un solo centro, de pacientes con cáncer de esófago en estadios I‐III que fueron tratados con quimiorradioterapia neoadyuvante y esofagectomía. Se analizaron, en cuatro momentos del tratamiento (desde el momento del diagnóstico hasta la quimiorradioterapia neoadyuvante, durante el tratamiento neoadyuvante, en los 30 días del período postoperatorio y a los 6 meses de la cirugía) las siguientes variables: VTE, tromboembolismo arterial, hemorragia grave, hemorragia no grave clínicamente relevante y mortalidad.

**Resultados:**

Se incluyeron 511 pacientes. La supervivencia a los 2 años fue del 67,3% (ic. del 95%, 63,2‐71,7). Durante el seguimiento de 2 años, 50 pacientes desarrollaron un VTE (9,8%), 20 un tromboembolismo arterial (3,9%), 21 hemorragias graves (4,1%) y 30 hemorragias no graves clínicamente relevantes (5,9%). El riesgo de estos accidentes fue notable en todas las etapas del tratamiento. A pesar de la profilaxis tromboembólica posquirúrgica, a los 30 días, 17 pacientes (3,3%) desarrollaron un VTE después de la operación. Los pacientes con VTE tuvieron una supervivencia menor (cociente de riesgos instantáneos, *hazard ratio* en función del tiempo 1,81; i.c. del 95%, 1,25‐2,64). La mayoría de los accidentes hemorrágicos ocurrieron en el contexto de una intervención médica y el 48% durante el uso concomitante de anticoagulación profiláctica o terapéutica.

**Conclusión:**

Los pacientes con cáncer de esófago tratados con quimiorradioterapia neoadyuvante y cirugía tienen un riesgo sustancial de sufrir accidentes tromboembólicos y hemorrágicos en todas las fases del tratamiento. La supervivencia es peor en aquellos pacientes que presentan accidentes tromboembólicos durante el seguimiento.

## Introduction

Patients with cancer have a fourfold to sevenfold increased risk of venous thromboembolism (VTE), comprising deep vein thrombosis (DVT) and pulmonary embolism (PE), compared with those without cancer^1^. Cancer‐associated VTE is a serious complication as it is associated with increased morbidity and mortality^2^, decreased quality of life^3^, and potential interruption or discontinuation of cancer treatment.

The incidence of VTE varies widely across tumour types. Patients with oesophageal cancer have a high risk of VTE, but the reported incidence is conflicting, ranging from 4 to 33 per cent^4^, ^5^, ^6^, ^7^. It is also unclear at which stage of treatment these patients are at highest risk of VTE. Although it has been reported that there is a considerable VTE risk during neoadjuvant chemotherapy^8^, there are no reports of the estimated risk for the interval from cancer diagnosis to initiation of chemotherapy and the period after surgery. Various types of cancer are associated with an increased risk of arterial thromboembolism^9^, but data for patients with oesophageal cancer are lacking. Besides the thrombotic risk, patients with cancer are also at increased risk of bleeding^1^ and this has not been investigated in detail in patients with oesophageal cancer.

Recently, two randomized placebo‐controlled trials^10^, ^11^ evaluated the use of direct anticoagulants to prevent cancer‐associated VTE in ambulatory patients classified as being at high risk of VTE based on the Khorana score^12^. Thromboprophylaxis was associated with a significant 4·1 per cent absolute reduction in the risk of VTE and a 1·0 per cent absolute increased risk of major bleeding^13^. Based on this positive benefit–harm ratio, several guidelines^14^, ^15^ now suggest thromboprophylaxis for 6 months in ambulatory patients with cancer who have a high‐risk Khorana score of at least 2 points. However, few patients with oesophageal cancer were included in these studies and outcomes were not reported according to tumour type. A risk estimation for thrombotic and bleeding risks in patients with oesophageal cancer is needed before starting thromboprophylaxis in these patients. The aim of this study was to assess the incidence of thromboembolic and bleeding events during the curative treatment and follow‐up of patients with oesophageal cancer, and to evaluate the association of these events with overall survival.

## Methods

This was a single‐centre, retrospective cohort study. Patients with stage I–III oesophageal adenocarcinoma or squamous cell carcinoma who received neoadjuvant chemoradiation and oesophagectomy with curative intent between August 2011 and May 2018 were identified from an institutional database. The Amsterdam University Medical Centre is a tertiary referral centre for patients with oesophageal cancer in the Netherlands. Patients were not eligible for curative treatment if they had an unresectable tumour (distant metastasis, supraclavicular lymph node involvement, or locally unresectable tumour or lymph nodes), were unfit for surgery, had a cervical tumour, or if life expectancy was less than 3 months^16^, ^17^. Exclusion criteria for this study were premalignant disease, anticoagulant therapy at cancer diagnosis, or ineligibility to receive curative treatment.

Neoadjuvant chemoradiation consisted of five cycles of paclitaxel and carboplatin with concurrent radiotherapy of 41·4 Gy administered in 23 fractions according to the CROSS (ChemoRadiotherapy for Oesophageal cancer followed by Surgery Study) regimen^18^. Some patients participated in a trial of neoadjuvant trastuzumab and pertuzumab (NCT02120911). Patients were scheduled for oesophagectomy 6–8 weeks after neoadjuvant chemoradiotherapy. Since 2009, most operations were performed by a minimally invasive approach (thoracolaparoscopic oesophagectomy). Patients did not undergo adjuvant therapy after oesophagectomy.

After surgery, patients received thromboprophylaxis with 2850 units nadroparin subcutaneously once daily for 30 days, in accordance with the protocol (Fraxiparin™; Aspen Pharma, Notre Dame De Bondeville, France). All patients had 4‐monthly follow‐up at the surgical outpatient clinic during the first 2 years after tumour resection^18^.

Original data were collected from the electronic patient records. The study was exempt from medical ethics committee approval, but was performed in accordance with local and national privacy legislation. Patients who declined to participate in medical research in general, as registered in the electronic patient record, were not included in the study. The study was registered at ClinicalTrials.gov (NCT03646409).

### Outcomes and follow‐up

Patients were followed from cancer diagnosis for a maximum of 2 years or until death, loss to follow‐up, or the end of data collection on 28 August 2018. Patients were regarded as lost to follow‐up if they did not visit the hospital in the 8 months before the last date of data collection and were not registered as deceased. The last contact date was used for these patients in the analyses. Main study outcomes were VTE, arterial thromboembolism, major bleeding and clinically relevant non‐major bleeding. Other study outcomes included cancer recurrence and all‐cause mortality. VTE was defined as any radiologically confirmed symptomatic or incidental PE, lower or upper extremity DVT, or splanchnic vein thrombosis. The event was considered incidental if diagnosed by an imaging test undertaken for reasons other than suspected VTE. Arterial thromboembolism was defined as the composite of ischaemic stroke, transient ischaemic attack, myocardial infarction and peripheral arterial occlusion/systemic embolism. Postoperative arterial splenic infarction was not considered an arterial thromboembolism as it is often caused by dividing the short gastric vessels during oesophagectomy. Major bleeding was defined according to the criteria of the International Society on Thrombosis and Haemostasis^19^ as bleeding leading to death, occurring in a critical location (such as intracranial), causing a drop in haemoglobin level of 2 g/l (1·24 mmol/l) or more, or requiring transfusion of 2 or more units of packed red blood cells. Major bleeding events were further classified based on the severity of clinical presentation using methods described previously^20^. Bleeding events that did not qualify as major bleeding, but that required any form of medical intervention or unscheduled medical contact, were considered clinically relevant non‐major bleeding events^19^. Study outcomes registered in other hospitals in between follow‐up visits were likely to be recorded by the authors' centre given the standard collection of clinical outcomes during all visits.

### Statistical analysis

Patient and tumour characteristics were summarized using standard descriptive statistics. Survival was calculated by the Kaplan–Meier method. Study outcomes were evaluated as proportions and as cumulative incidences using the cumulative incidence function in which death unrelated to the study outcome was treated as a competing risk. Outcomes were estimated at 6, 12 and 24 months of follow‐up. Outcomes were also evaluated during all clinically relevant treatment stages: from cancer diagnosis to the start of chemoradiotherapy, during neoadjuvant treatment, in the 30 days after oesophagectomy, and during the 6‐month interval following this postoperative period. The association between potential risk factors and VTE or major bleeding during the first 6 months was explored by computing univariable subdistribution hazard ratios (SHRs) with 95 per cent confidence intervals using the Fine and Gray competing‐risk regression model^21^. The association between VTE or arterial thromboembolism during follow‐up and survival was assessed by regarding these events as time‐varying co‐variate adjusted for age, cancer stage, disease progression and disease relapse. The Khorana VTE risk score was calculated for all patients, assigning 0 points to oesophageal cancer, as in the derivation study^12^. In a sensitivity analysis, the score was calculated by assigning 2 points for oesophageal cancer, similar to gastric cancer in the original score. The sample size was based on all patients who underwent curative tumour resection in this hospital. Statistical analyses were performed using R software version 3.5.1 (R Project for Statistical Computing, Vienna, Austria), specifically using the cmprsk package.

## Results

A total of 511 patients were included, with a median age of 64 (i.q.r. 58–70) years; 78·3 per cent were men (*Table* 1). The exact disease stage could not be determined for 16 patients (3·1 per cent) because of an obstructing tumour, but none of the patients had metastatic disease (stage IV). The majority of oesophageal cancers were adenocarcinomas (77·9 per cent). The survival rate at 24 months of follow‐up was 67·3 (95 per cent c.i. 63·2 to 71·7) per cent. Three patients (0·6 per cent) were lost to follow‐up by 24 months.

**Table 1:** Patient characteristics

	No. of patients* (*n* = 511)
**Age (years)** †	64 (58–70)
**Sex ratio (F** : **M)**	111 : 400
**BMI (kg/m** ^**2**^ **)** †	25·4 (22·6–28·4)
**ECOG performance status**	
0	313 (61·3)
1	107 (20·9)
2	9 (1·8)
Missing	82 (16·0)
**Clinical disease stage**	
I	36 (7·0)
II	145 (28·4)
III	314 (61·4)
Not determined	16 (3·1)
**Tumour histology**	
Adenocarcinoma	398 (77·9)
Squamous cell carcinoma	110 (21·5)
Other	3 (0·6)
**Central venous catheter during follow‐up**	41 (8·0)
**Cancer treatment**	
CROSS*	487 (95·3)
CROSS + neoadjuvant trastuzumab and pertuzumab	24 (4·7)
**Interval from cancer diagnosis to start of chemoradiotherapy (days)** †	29 (25–35)
**Interval from cancer diagnosis to tumour resection (days)** †	125 (113–141)
**Medication use at baseline**	254 (49·7)
Anticoagulant	0 (0)
Antiplatelet	87 (17·0)
Proton pump inhibitor	211 (41·3)
NSAIDs (≥ 4 days per week)	8 (1·6)
**Prechemotherapy laboratory results** †	
Haemoglobin (mmol/l)	8·9 (8·3–9·4) (*n* = 482)
Leucocyte count (/mm^3^)	8·0 (6·7–9·5) (*n* = 476)
Platelet count (/mm^3^)	266 (222–319) (*n* = 478)
Creatinine (μg/l)	78 (68–89) (*n* = 477)
**Surgical approach**	
Thoracolaparoscopic	405 (79·3)
Open	91 (17·8)
Hybrid	15 (2·9)

* With percentages in parentheses unless indicated otherwise;

† values are median (i.q.r.).

‡ Anticancer therapy according to CROSS consisted of neoadjuvant intravenous carboplatin, paclitaxel and concurrent radiotherapy. ECOG, Eastern Cooperative Oncology Group; NSAID, non‐steroidal anti‐inflammatory drug.

### Thromboembolic events

Some 32 of 511 patients (6·3 per cent) developed VTE in the first 6 months, 40 (7·8 per cent) in the first 12 months and 50 (9·8 per cent) during the 24 months of follow‐up (*Table* 2). Of 50 events by 24 months, 16 were detected incidentally and 34 were symptomatic. Thirty‐two events were PE, eight were DVT of the lower extremities, four (8 per cent) DVT of the upper extremities, one PE and concurrent DVT, three were abdominal, and two occurred at another location (*Table* 3).

**Table 2:** Risk of thromboembolic and bleeding events

	Venous thromboembolism	Arterial thromboembolism	Major bleeding	Clinically relevant non‐major bleeding
**Time after cancer diagnosis (months)**				
6	32 (6·3)	14 (2·7)	16 (3·1)	17 (3·3)
12	40 (7·8)	18 (3·5)	19 (3·7)	22 (4·3)
24	50 (9·8)	20 (3·9)	21 (4·1)	30 (5·9)
**Treatment stage**				
From cancer diagnosis to start of chemoradiotherapy	1 (0·2)	3 (0·6)	2 (0·4)	3 (0·6)
Neoadjuvant stage: from start of chemoradiotherapy until surgery	13 (2·5)	3 (0·6)	3 (0·6)	4 (0·8)
Postoperative stage: from hospital admission for oesophagectomy until postoperative day 30	17 (3·3)	8 (1·6)	12 (2·3)	6 (1·2)
6‐month interval after postoperative stage	8 (1·6)	4 (0·8)	3 (0·6)	10 (2·0)

Values in parentheses are percentages. Only first events are reported for each patient.

**Table 3:** Details of venous and arterial thromboembolic events

	Time after cancer diagnosis	
	6 months	24 months
**Venous thromboembolic events**	32	50
Symptomatic	21	34
Incidental	11	16
Location		
PE	24	32
DVT, lower extremities	3	8
PE and DVT	1	1
DVT, upper extremities	2	4
Abdominal	1	3
Other location	1	2
**Arterial thromboembolic events**	14	20
Kidney	0	2
Spleen and liver	2	3
Stroke	4	6
Liver	0	1
Myocardial infarction	3	3
Peripheral artery occlusion	5	5

PE, pulmonary embolism; DVT, deep vein thrombosis.

Of the 50 patients with VTE, ambulant anticoagulation was initiated in 46; seven patients needed hospital admission and six were admitted to intensive care, but VTE was not the main reason for this in some patients. An inferior vena cava filter was inserted in three patients to prevent PE during surgery. Nine of 16 patients with an incidental VTE had segmental or central PE, three abdominal VTE, two subsegmental PE and two upper extremity VTE. Anticoagulation was initiated in 13 of 16 patients with incidental VTE.

Arterial thromboembolism occurred in 14 patients (2·7 per cent) by 6 months, in 18 (3·5 per cent) by 12 months and in 20 (3·9 per cent) by 24 months of follow‐up (*Table* 2). Arterial thromboembolic events included ischaemic stroke (6 patients), peripheral artery occlusion (5), myocardial infarction (5) and other arterial thromboembolic events (6). Characteristics of arterial thromboembolic events are shown in *Table* 3.

**Figure 1:**
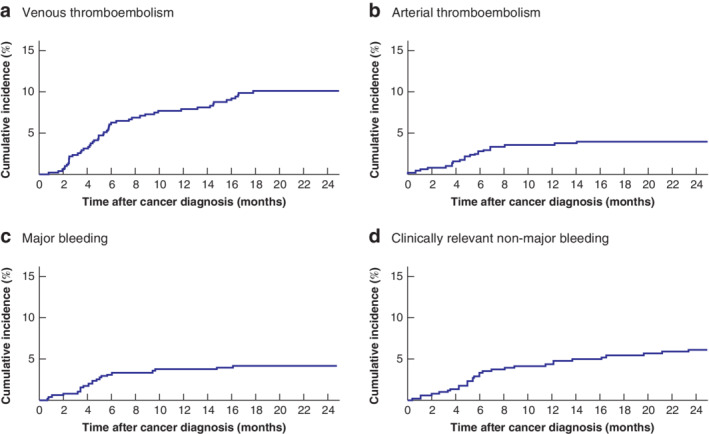
Cumulative incidence of venous thromboembolism, arterial thromboembolism, major bleeding and clinically relevant non‐major bleeding during 24‐month follow‐up: competing‐risk analysis **a** Venous thromboembolism, **b** arterial thromboembolism, **c** major bleeding and **d** clinically relevant non‐major bleeding.

Cumulative incidence curves for VTE and arterial thromboembolism, in which death was treated as a competing risk, are shown in *Fig*. 1 *a*,*b*.

**Figure 2:**
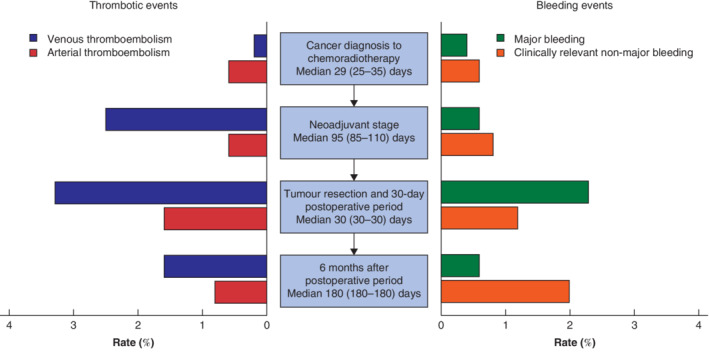
Proportion with thromboembolic and bleeding events during consecutive treatment stages among patients with oesophageal cancer undergoing treatment with curative intent Values in parentheses are interquartile range.

In the analysis in which VTE was treated as a time‐varying co‐variate, VTE was associated with increased mortality (hazard ratio (HR) 1·81, 95 per cent c.i. 1·25 to 2·64). The corresponding HR adjusted for age, cancer stage, disease progression and disease relapse was 2·13 (1·46 to 3·12). Similarly, arterial thromboembolism was associated with increased mortality (HR 2·90, 1·75 to 4·83; adjusted HR 2·90, 1·74 to 4·85).

### Bleeding events

Major bleeding occurred in 21 patients (4·1 per cent) by 24 months of follow‐up (*Table* 2). Nine of the 21 major bleeding events were gastrointestinal, two urogenital and ten occurred at another location. About half were classified as category 3 or 4, which is considered a severe clinical presentation of a major bleed. Two‐thirds of the major bleeding events occurred during or shortly after interventions. Details of major bleeding events, including use of anticoagulant therapy at the time of bleeding, are shown in *Table* 4. Clinically relevant non‐major bleeding occurred in 30 patients (5·9 per cent) by 24 months of follow‐up (*Table* 2). Details of non‐major bleeding events are shown in *Table* 5.

**Table 4:** Details of major bleeding events

	Time after cancer diagnosis	
	6 months (*n* = 16)	24 months (*n* = 21)
**Location**		
Gastrointestinal	9	9
Urogenital	2	2
Other	5	10
Severity classification of major bleeding*		
1	2	2
2	9	9
3	4	7
4	1	3
**ISTH major bleeding criteria†**		
Critical location	1	1
Fatal bleed	1	5
Haemoglobin drop ≥ 2 g/dl	14	15
Transfusion of ≥ 2 units blood	2	2
**Anticoagulant being used at time of bleeding**		
Prophylactic LMWH	5	5
Therapeutic LMWH	2	3
Direct oral anticoagulants	0	0
Vitamin K antagonist	1	2
No anticoagulant	8	11
**Following medical procedure**	12	14
Perioperative, during tumour resection	2	2
30‐day postoperative period after tumour resection	10	10
Following decannulation tracheostomy	0	1
Following tracheostomy	0	1
**Not following medical procedure**	4	7
Spontaneous upper gastrointestinal bleed	4	4
Bleeding from open chest wound	0	1
Bleeding from fistula‡	0	2

* Classification of Bleker *et al*.^20^. Category 1: bleeding events without any clinical emergency; category 2: all bleeding events that could not be classified into any of the other three categories, as they needed some measures but without clear urgency; category 3: bleeding events with great medical emergency, such as haemodynamic instability, or cerebral bleeding with neurological symptoms; category 4: bleeding events that are fatal before or almost immediately on entering hospital.

† Patients could fulfil multiple International Society on Thrombosis and Haemostasis (ISTH) criteria for major bleeding.

‡ Bronchopleural fistula (1), fistula between aorta and gastric tube (1). LMWH, low molecular weight heparin.

**Table 5:** Details of clinically relevant non‐major bleeding events

	Time after cancer diagnosis	
	6 months (*n* = 17)	24 months (*n* = 30)
**Location**		
Gastrointestinal	7	15
Epistaxis	3	4
Pulmonary	2	2
Other location	5	9
**Anticoagulant being used at time of bleeding**		
Prophylactic LMWH	5	8
Therapeutic LMWH	1	2
Direct oral anticoagulants	0	1
Vitamin K antagonist	0	0
No anticoagulant	11	19

LMWH, low molecular weight heparin.

The cumulative incidences of major and clinically relevant non‐major bleeding events estimated by competing‐risk analysis are shown *Fig*. 1 *c*,*d*.

### Treatment stages

The median interval from cancer diagnosis to initiation of chemoradiation was 29 (i.q.r. 25–35) days. In this stage, one patient (0·2 per cent) developed VTE, three (0·6 per cent) arterial thromboembolism, two (0·4 per cent) had major bleeding and three (0·6 per cent) had clinically relevant non‐major bleeding (*Table* 2 and *Fig*. 2).

The median interval between the start of neoadjuvant treatment and surgery was 95 (85–110) days. During this neoadjuvant stage, 13 patients (2·5 per cent) developed VTE, three (0·6 per cent) arterial thromboembolism, three (0·6 per cent) had major bleeding and four (0·8 per cent) experienced clinically relevant non‐major bleeding. Time to tumour resection was longer for patients with a VTE at this stage than in those without (median 99 *versus* 95 days), although this was not significant (*P* = 0·903).

In the 30 days after oesophagectomy, VTE occurred in 17 patients (3·3 per cent), arterial thromboembolism in eight (1·6 per cent), major bleeding in 12 (2·3 per cent) and clinically relevant non‐major bleeding in six (1·2 per cent). Prophylactic low molecular weight heparin (LMWH) was used by 14 of 17 patients with VTE in this period, and by all patients with arterial thromboembolism. Twelve of the 17 VTE events coincided with other postoperative complications, including anastomotic leak (5), acute respiratory distress syndrome (2) and pneumonia (2). Two of 12 major bleeding events occurred during surgery. Details of major bleeding events are shown in *Table S1* (supporting information).

### Risk factors for venous thromboembolism and major bleeding

Of the risk factors evaluated, BMI (SHR per kg/m^2^: 1·08, 95 per cent c.i. 1·02 to 1·15) and Eastern Cooperative Oncology Group (ECOG) performance score (SHR for score 1 *versus* 0: 0·24, 0·06 to 0·99) were significantly associated with 6‐month VTE risk in univariable analyses (*Table S2*, supporting information). The Khorana score was 2 points or less for 507 patients (99·2 per cent; low to intermediate risk) and 3 or more for four patients (0·8 per cent; high risk). None of the four patients classified as high risk developed VTE. In the sensitivity analysis in which a Khorana score of 2 points was assigned for oesophageal cancer, 384 patients (75·1 per cent) had a score of 2 or lower and 127 (24·9 per cent) a score of at least 3. In both risk groups, 6·3 per cent of patients developed VTE in the first 6 months after cancer diagnosis (SHR 1·24, 95 per cent c.i. 0·57 to 2·70). Applying the recently proposed lower threshold of 2 points or more, 491 patients (96·1 per cent) had a low‐risk score (1 point or less) and 20 (3·9 per cent) a high‐risk score (2 points or more). Again, none of the patients in the high‐risk group developed VTE during follow‐up.

Haemoglobin concentration below 6·2 mmol/l (SHR 0·63, 0·41 to 0·98) and a leucocyte count exceeding 11 per mm^3^ (SHR 1·15, 1·08 to 1·22) determined before start of chemoradiotherapy were associated with major bleeding during the first 6 months (*Table S1*, supporting information).

## Discussion

In this study, the risk of thromboembolic and bleeding events was considerable in patients with oesophageal cancer receiving treatment with curative intent. By 24 months of follow‐up, VTE had been diagnosed in 9·8 per cent of patients, arterial thromboembolism in 3·9 per cent, major bleeding in 4·1 per cent and clinically relevant non‐major bleeding in 5·9 per cent. During follow‐up, 21·1 per cent of patients were affected by any of these complications. This risk was present during all treatment phases, including the time from cancer diagnosis to first chemoradiation and the neoadjuvant treatment stage, but was highest in the 30 days after surgery. During this postoperative stage, the absolute risk of VTE was 3·3 per cent and that of arterial thromboembolism 1·6 per cent, despite the use of thromboprophylaxis in most patients.

VTE and arterial thromboembolism are associated with short‐ and long‐term morbidity^3^, which is increasingly important as new treatment regimens for oesophageal cancer have improved survival^22^. In addition, thromboembolic complications may lead to a delay or interruption of cancer treatment. Patients included in this study who developed such a complication in the neoadjuvant stage indeed had a longer interval to tumour resection, but this difference was small (5–6 days) and not statistically significant. VTE and arterial thromboembolism were associated with worse survival in this study.

Thromboprophylaxis with direct anticoagulants during the first 6 months after initiation of chemotherapy is suggested nowadays in ambulatory patients with a high‐risk Khorana score^14^, ^15^. This recommendation is based on two RCTs^10^, ^11^ that evaluated 6 months of half‐therapeutic dose apixaban or rivaroxaban. Notably, these studies included only a few patients with oesophageal cancer and most of the enrolled patients had advanced disease. The results of the present study suggest that thromboprophylaxis should not be considered in patients with oesophageal cancer who undergo neoadjuvant chemoradiation followed by surgery. From cancer diagnosis to surgery, the combined risk of VTE and arterial thromboembolism was about 4 per cent in 4 months, or 1 per cent per month. In general, this risk is not perceived to be sufficiently high to justify thromboprophylaxis. For example, in the SAVE‐ONCO trial^23^ of patients with advanced cancer, the risk of VTE was 1·2 and 3·4 per cent in the semuloparin (LMWH) and placebo groups respectively (median follow‐up 3·5 months). The corresponding number needed to treat of 45 did not result in a recommendation to provide universal thromboprophylaxis. Moreover, in the present study, the risk of clinically relevant bleeding was substantial in the interval from cancer diagnosis to surgery, which warrants an even more conservative approach. There was no benefit of using the Khorana score to identify a high‐risk group, as the risk was similar or lower in those with a high‐risk score.

International guidelines^24^ recommend that patients who undergo major abdominal cancer surgery should receive thromboprophylaxis for 4 weeks after surgery. Patients in the present study received thromboprophylaxis with 2850 units nadroparin once daily for 30 days after operation. However, the combined risk of VTE and arterial thromboembolism was about 5 per cent during this period. This risk is in line with previous studies^25^, ^26^ that reported a substantial residual VTE risk in patients with abdominal cancer despite thromboprophylaxis. A potential explanation is that the prophylactic dose of 2850 units nadroparin is insufficient. Non‐compliance with 4 weeks of thromboprophylaxis may also play a role as it is well recognized that daily subcutaneous injections are burdensome for many patients. These observations indicate that clinicians should inform their patients carefully about the importance of measures to prevent VTE. Whether direct oral anticoagulants are also effective and safe in preventing thromboembolism after surgery in patients with cancer, with improved compliance, deserves further study. A possible drawback of direct oral anticoagulants is the increased bleeding tendency in patients with gastrointestinal cancer^27^, ^28^, ^29^.

Few other studies have evaluated thromboembolic complications in patients with oesophageal cancer. A systematic review and meta‐analysis^8^ including six retrospective cohort studies reported a pooled incidence of symptomatic or incidental VTE of 7 per cent during neoadjuvant chemoradiation. The lower incidence of 2·5 per cent in the present study may be explained by differences in case mix, definitions of outcome and duration of study. Papaxoinis and colleagues^30^ reported an incidence of VTE and arterial thromboembolism of 9 and 4 per cent respectively in a cohort of 590 patients with oesophagogastric cancer, with a median follow‐up of 43 months. More than half of the events were detected incidentally.

Some studies have reported that a substantial number of adverse events occur within 90 days after surgery^31^. In the present study, the cumulative incidences in the 90‐day period were only increased marginally compared with those after 30 days: 3·9 per cent for VTE, 2·2 per cent for arterial thromboembolism, 2·7 per cent for major bleeding and 2·4 per cent for clinically relevant non‐major bleeding.

BMI and ECOG performance status were associated with the 6‐month VTE risk. Both have been recognized previously as risk factors for VTE in patients with cancer^12^, ^32^. The Khorana VTE risk score was not, in contrast to findings of other cohort studies^33^. It is unclear whether this discrepancy is caused by a difference in case mix because only patients receiving neoadjuvant chemoradiotherapy were included here, or simply because the Khorana risk score may not predict VTE in this patient group. A low haemoglobin level and high leucocyte count before the start of chemoradiotherapy were associated with the 6‐month risk of major bleeding. Anaemia is a well known predictor of bleeding^34^. The reason for the association between leucocyte count and bleeding is unclear, but leucocytosis may just be a reflection of more aggressive tumours that are more likely to bleed^35^.

Strengths of this study include the large, homogeneous cohort of patients who received similar treatment and underwent regular follow‐up. The cumulative incidence estimated by the competing‐risk approach accounts for the concurrent risk of death and minimizes overestimation of study outcomes. Several limitations also need to be taken into account. Owing to the retrospective nature of this study, some outcomes may have been missed, potentially leading to lower estimates. However, the routine follow‐up of patients after curative treatment minimizes this risk; loss to follow‐up at 24 months was just 0·6 per cent. The present study outcomes are not applicable to all patients with oesophageal cancer as only those deemed eligible for oesophagectomy were included. Moreover, only patients who underwent surgery were included, introducing immortal time bias which may have led to conservative estimates. The results are potentially not applicable to patients with oesophageal cancer receiving other treatments, such as FLOT (5‐fluorouracil, leucovorin, oxaliplatin, docetaxel) chemotherapy^36^ or non‐minimally invasive surgery.

This study has demonstrated a substantial thromboembolic risk in patients with oesophageal cancer receiving treatment with curative intent. The absolute risk between cancer diagnosis and surgery was, however, too low to justify routine thromboprophylaxis, especially given the concurrent considerable risk of bleeding complications at this stage. During the 30 days after resection of oesophageal cancer, the risk of VTE and arterial thromboembolism was high, despite thromboprophylaxis. Whether direct oral anticoagulants would be more effective than LMWH during this period, or would perhaps lead to better compliance, deserves further study. As the Khorana score was unable to discriminate between patients at low or high risk of VTE, new risk factors for thromboembolic and bleeding complications need to be identified in order to better select patients for thromboprophylaxis, and avoid it in patients with a high bleeding risk.

## Supporting information


**Appendix S1**: Supporting information
