# Supplementary material for: Thromboembolic and bleeding complications in patients with oesophageal cancer
Source: Br J Surg. 2020 May 19;107(10):1324–33. doi: 10.1002/bjs.11665 (PMC7497123; doi:10.1002/bjs.11665)
Supplement: Supplementary file 1 — Appendix S1: Supporting information [file BJS-107-1324-s001.docx]

**BJS11665**

# **Thromboembolic and bleeding complications in patients with oesophageal cancer**

F. I. Mulder, A. Hovenkamp, H. W. M. van Laarhoven, H. R. Büller, P. W. Kamphuisen, M. C. C. M. Hulshof, M. I. van Berge Henegouwen, S. Middeldorp and N. van Es

**Table S1** **Specification of major bleeding events during oesophagectomy and 30-days postoperatively.***

| **Location** | **Anticoagulation at time of bleeding event** |
| --- | --- |
| **Intra-operative major bleeding events** | |
| Heavy blood loss from splenic artery during surgery. Estimated blood loss 3 litre. Patient admitted to intensive care unit, and received transfusions of fresh frozen plasma, platelets, and red blood cells. | **-** |
| Heavy blood loss from splenic artery right after surgery. Artery surgically closed during re-operation. | **-** |
| **Major bleeding events in the 30-days following surgery** | |
| Urogenital bleeding 9 days after surgery after removal of urinary catheter. | Therapeutic anticoagulation |
| Bleeding from peptic ulcer while using prophylactic fraxiparin. | Thromboprophylaxis |
| Urogenital bleeding while using urinary catheter. | Therapeutic anticoagulation |
| Fatal hematemesis 21 days after surgery. | Thromboprophylaxis |
| Active blood loss at anastomotic line 18 days after surgery. | Thromboprophylaxis |
| Hematemesis 15 days after surgery. | Thromboprophylaxis |
| Local blood loss after removing thorax drains. | Thromboprophylaxis |
| Rectal blood loss caused by ischemic mucosal tissue at terminal ileum. | Therapeutic anticoagulation |
| Local blood loss after removing thorax drains. | Thromboprophylaxis |
| Local blood loss after removing thorax drains. | Therapeutic anticoagulation |

* Major bleeding was defined according to the criteria of the International Society on Thrombosis and Haemostasis (ISTH) as bleeding (1) leading to death, (2) occurring in a critical location (e.g. intracranial), (3) causing a haemoglobin drop of 2 g/L or more (1.24 mmol/L), or (4) requiring transfusion of 2 or more units of packed red blood cells.

**Table S2 The association between risk factors and venous thromboembolism and major bleeding during 6 months follow-up after cancer diagnosis.**

| **Variable** | | **SHR venous thromboembolism**  **(95% CI)** | **SHR major bleeding**  **(95% CI)** |
| --- | --- | --- | --- |
| Age, per year increase | | 1.01 (0.98-1.04) | 0.98 (0.90-1.06) |
| Female | | 1.04 (0.45-2.40) | 0.83 (0.24-2.91) |
| Body mass index | |  |  |
|  | Per kg/m^2^ increase | **1.08 (1.02-1.15)** | 1.06 (0.97-1.16) |
|  | ≥ 35 kg/m^2^ | 2.17 (0.49-9.74) | 4.25 (0.99-18.17) |
| Pre chemotherapy creatinine clearance | |  |  |
|  | Per increase in ml/minute | 1.01 (0.99-1.03) | 1.01 (0.98-1.04) |
|  | <60 ml/minute | 0.49 (0.12-2.05) | NA - no events |
| Pre chemotherapy haemoglobin | |  |  |
|  | Per increase in mmol/l | 1.01 (0.70-1.46) | **0.63 (0.41-0.97)** |
|  | <6.2 mmol/l | NA - no events | **10.55 (3.11-35.79)** |
| Pre chemotherapy leukocyte count | |  |  |
|  | Per mm^3^ increase | 0.88 (0.74-1.05) | **1.15 (1.08-1.22)** |
|  | >11 per mm^3^ | 0.49 (0.12-2.05) | **5.15 (1.89-14.07)** |
| Pre chemotherapy platelet count | |  |  |
|  | Per 10 mm^3^ increase | 0.99 (0.95-1.03) | 1.04 (0.99-1.09 |
|  | ≥ 350 per mm^3^ | 0.94 (0.33-2.65) | 0.90 (0.21-3.90) |
| Prior venous thromboembolism | | 2.63 (0.33-21.24) | - |
| History of cardiovascular disease | | 0.94 (0.36-2.43) | 1.14 (0.32-4.02) |
| Use of antiplatelet therapy | | 0.93 (0.36-2.43) | 0.70 (0.16-3.08) |
| ≥3 alcoholic units per day | | 0.78 (0.38-1.59) | 1.70 (0.55-5.28) |
| Khorana score with 0 points for tumour type | |  |  |
|  | Per point increase of score | 0.97 (0.58-1.65) | - |
|  | 3 vs lower | NA - no events | - |
|  | 2 vs lower | NA - no events | - |
| Khorana score with 2 points for tumour type | |  |  |
|  | Per point increase of score | 0.97 (0.58-1.63) | - |
|  | 3 vs lower | 1.24 (0.57-2.70) | - |
| Squamous cell carcinoma vs adenocarcinoma | | 0.87 (0.36-2.11) | 1.20 (0.39-3.70) |
| Tumour histopathological differentiation | |  |  |
| Well-differentiated | | Reference | Reference |
|  | Moderately | 2.25 (0.30-17.11) | 1.56 (0.20-12.41) |
|  | Poor | 2.95 (0.38-22.72) | 1.29 (0.15-11.22) |
| Disease stage at baseline | |  |  |
|  | Stage I | Reference | Reference |
|  | Stage II | 0.58 (0.15-2.26) | NA - no events |
|  | Stage III | 0.73 (0.22-2.50) | NA - no events |
| Neoadjuvant therapy with trastuzumab and pertuzumab | | 0.65 (0.09-4.62) | 1.33 (0.18-9.74) |
| Currently smoking vs. never smoked | | 0.65 (0.26-1.58) | 0.94 (0.27-3.34) |
| ECOG performance status | |  |  |
| 0 | | Reference |  |
|  | 1 | **0.24 (0.06-0.99)** | 0.78 (0.23-2.86) |
|  | 2 | NA - no events | NA - no events |

Abbreviations: CI, confidence interval; ECOG, Eastern Cooperative Oncology Group; kg, kilogram; l, litre; mm, millimetre; SHR, subdistribution hazard ratio.
